# Functional language assessment in children with autism spectrum disorder in a Portuguese and English language context

**DOI:** 10.1192/j.eurpsy.2025.1101

**Published:** 2025-08-26

**Authors:** L. D. Bezerra, C. J. S. Ribeiro, C. A. D. L. H. Amato

**Affiliations:** 1 Universidade Presbiteriana Mackenzie, São Paulo, Brazil

## Abstract

**Introduction:**

Autism spectrum disorder (ASD) presents specific characteristics in child development. Behavioral and, mainly, communicative aspects are the most frequent for diagnostic closure (APA Artmed 2014; Asperger Archiv F. Psychiatrie 1944; 117 76-136). The absence, delay or difficulties in establishing expressive language are important social markers in early childhood, being one of the main reasons that lead families to seek therapeutic guidance (Amato USP 2006; Andrade UFPB 2017; Andrade Pró-Fono 2023). One of the aspects related to the communication of children with ASD is the interest in foreign languages, which manifests itself from early childhood (Avelar Universidade Presbiteriana Mackenzie 2018). The use of a foreign language for interpersonal communication presented spontaneously, by children with ASD, is something that needs to be evaluated within the functional aspects of social communication, since the major communicative difficulties faced are related to pragmatic aspects (Andrade Pró-Fono 2023; Balestro *et al*. Rev Soc Bras Fonoaudiol 2012; 17 279-86). The form and means of communication, length of permanence and communicative quality are fundamental elements to be analyzed when comparing expected child development to chronological age. In a research process involving humans, it makes perfect sense to anticipate and understand linguistic, cultural and educational differences, in order to avoid contextual and communicative variables that conflict with the proposed aims.

**Objectives:**

The objective of this study is to evaluate the functional communication of children with ASD in Portuguese and English language contexts.

**Methods:**

16 children were selected, aged 42 to 72 months, without relatives and/or living with multilingual people. Samples were collected by recording spontaneous communication between patient and therapist in a natural intervention environment. 5-minute clips of greater interaction were made and, from these, the analyzes were carried out. This research has an opinion approved by the Ethics Committee, under number 6,890,786.

**Results:**

Parents’ perception of their children’s communication difficulties were unanimously reported. Greater communicative ability was demonstrated in the Portuguese language, however, communicative ability and functionality was verified in the English language, without significant statistical differences when comparing the two languages. In the production of grammatical classes, there was greater production in the Portuguese language, with greater lexical production.

**Image 1:**

**Figure:**
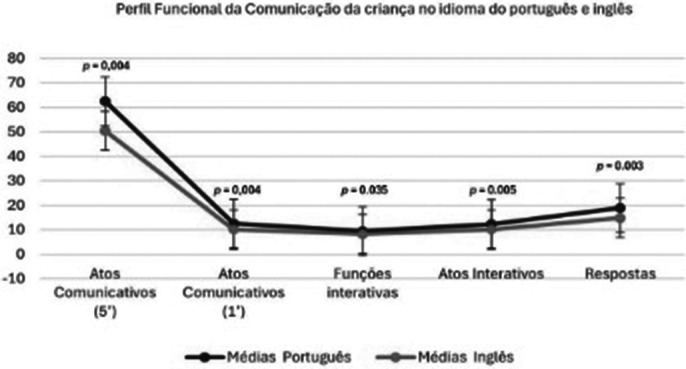

**Conclusions:**

The children under observation and evaluation in this study showed satisfactory correlation and performance in the proposed tests. There was communicative performance, with results without significant statistical differences in the Functional Communication Profile in both languages. More studies will be needed to support these results.

**Disclosure of Interest:**

None Declared

